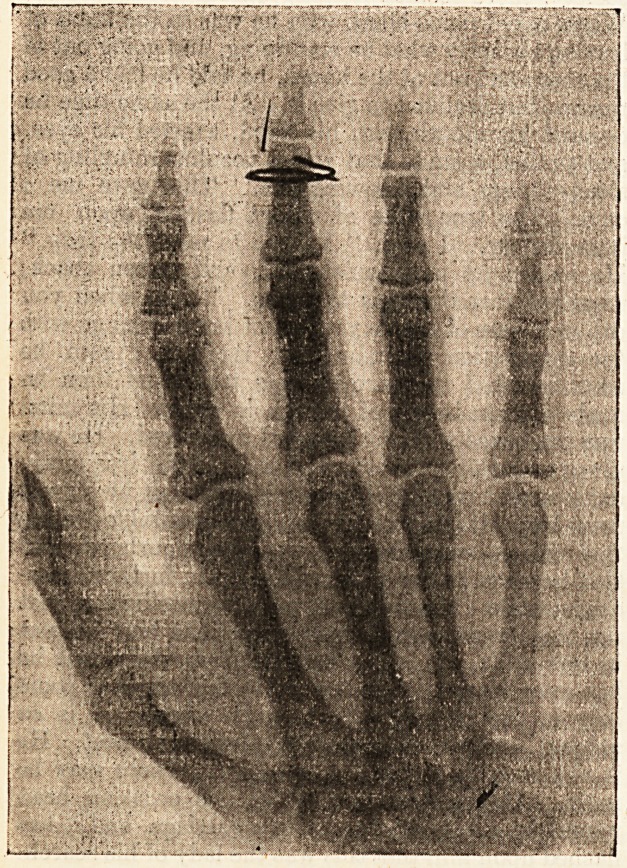# Practical Hints for X-Ray Work

**Published:** 1908-03-21

**Authors:** 


### Resident Medical Officers' Department.

### PRACTICAL HINTS FOR X-RAY WORK.

Now that the $x$-rays have become so universally used it often happens in some of the provincial hospitals that the management of this department falls to the lot of the house surgeon. In this short article I wish to mention one or two points which may chance to be of use to those who read them. I do not in any way claim originality for any of them.

I have found in searching for needles that an $x$-ray plate taken in the following way is a great help:—The patient can generally show you the point of entry of the needle; this being so, take a piece of wire, and encircle the limb or finger with it, so that the wire lies directly over the wound of inlet, twist off the free ends, making them lie over the opposite side of the limb. Now proceed to take your picture on general principles. The negative will show you the shadow of the wire as well as that of the needle (as shown in illustration). In this way you can see whether the needle is above or below the point of entry. Furthermore, the wire nearest the plate will give a more defined shadow than that on the far side of the limb where the wire has been twisted off. If you now compare this shadow with that of the needle you may be able to get some idea as to the depth at which it is lying.

In taking pictures of tuberculous limbs, which may have sinuses into which iodoform emulsion has been injected, it is well to remember that the iodoform throws a shadow on the plate, giving the appearance of an area of rarified bone. It sometimes happens that, owing to a fault of the tube or the coil, to get a good result a long exposure is necessary. The exposure may be shortened by at least a quarter by placing a fluorescent screen on the plate, and allowing the patient's limb to rest on this. The majority of these screens are composed of parchment stretched on a frame coated with glue, and covered with barium-platinum-cyanide. In some cases the negative presents a granular appearance, owing to the large size of the crystals. If a tungstate of calcium screen is used this granular appearance is absent; they are less expensive, but are not so good for direct observation.

## Figures and Tables

**Figure f1:**